# Current ecological understanding of fungal-like pathogens of fish: what lies beneath?

**DOI:** 10.3389/fmicb.2014.00062

**Published:** 2014-02-19

**Authors:** Rodolphe E. Gozlan, Wyth L. Marshall, Osu Lilje, Casey N. Jessop, Frank H. Gleason, Demetra Andreou

**Affiliations:** ^1^Unité Mixte de Recherche Biologie des Organismes et Écosystèmes Aquatiques (IRD 207, CNRS 7208, MNHN, UPMC), Muséum National d'Histoire NaturelleParis Cedex, France; ^2^Centre for Conservation Ecology and Environmental Sciences, School of Applied Sciences, Bournemouth UniversityPoole, Dorset, UK; ^3^BC Centre for Aquatic Health SciencesCampbell River, BC, Canada; ^4^School of Biological Sciences, University of SydneySydney, NSW, Australia

**Keywords:** emerging infectious disease, aquatic, extinction, vertebrate, global, biodiversity, Oomycota, Mesomycetozoea

## Abstract

Despite increasingly sophisticated microbiological techniques, and long after the first discovery of microbes, basic knowledge is still lacking to fully appreciate the ecological importance of microbial parasites in fish. This is likely due to the nature of their habitats as many species of fish suffer from living beneath turbid water away from easy recording. However, fishes represent key ecosystem services for millions of people around the world and the absence of a functional ecological understanding of viruses, prokaryotes, and small eukaryotes in the maintenance of fish populations and of their diversity represents an inherent barrier to aquatic conservation and food security. Among recent emerging infectious diseases responsible for severe population declines in plant and animal taxa, fungal and fungal-like microbes have emerged as significant contributors. Here, we review the current knowledge gaps of fungal and fungal-like parasites and pathogens in fish and put them into an ecological perspective with direct implications for the monitoring of fungal fish pathogens in the wild, their phylogeography as well as their associated ecological impact on fish populations. With increasing fish movement around the world for farming, releases into the wild for sport fishing and human-driven habitat changes, it is expected, along with improved environmental monitoring of fungal and fungal-like infections, that the full extent of the impact of these pathogens on wild fish populations will soon emerge as a major threat to freshwater biodiversity.

## Introduction

Fishes are susceptible to diseases caused by a large number of infectious agents including viruses, bacteria, true fungi, fungal-like microrganisms, other protists, and metazoans. This review will briefly discuss true fungal pathogens and then focus on commonly reported zoosporic and amoeboid fungal-like pathogens in the Oomycota and Mesomycetozoea. In general, the number of reported fungal and fungal-like pathogens responsible for diseases in animals is on the increase globally (Fisher et al., 2009; Holdich et al., 2009; Loo, 2009; Frick et al., 2010; Ratnieks and Carreck, 2010; Sarmiento-Ramírez et al., 2010). As such, they are truly emerging diseases with increasing incidence, geographic range, virulence, and some of these fungal and fungal-like pathogens have recently been found in new hosts or are newly discovered (Berger et al., 1998; Brown, 2000; Daszak et al., 2000; Kim and Harvell, 2004; Blehert et al., 2009; Peeler et al., 2010; Cameron et al., 2011). The underpinning drivers of this observed increase remain unclear but these pathogens are known to be opportunistic (Fisher et al., 2012), to have resilient and relatively long-lived environmental stages (Mitchell et al., 2008; Andreou et al., 2009) and may have benefited from recent increase in global trade (Brasier, 2008) and spread of invasive species (Gozlan et al., 2010). Thus increasingly infectious outbreaks are reported in a broad range of species from coral (Kim and Harvell, 2004) to wheat (Wanyera et al., 2006); notable examples include local extinctions of bats (Frick et al., 2010), bees (Ratnieks and Carreck, 2010), turtles (Sarmiento-Ramírez et al., 2010), amphibians (Fisher et al., 2009) and fish (Gozlan et al., 2005, 2009). In aquatic ecosystems fungi and fungal-like pathogen detection in fish hosts is more complicated due to the lack of direct observation of their hosts contrary to frogs or coral, for example (Gozlan, 2012). This is particularly true in freshwater systems where, despite being responsible for pan-continental population extinctions, some diseases caused by fungal and fungal-like pathogens are chronic with no clear external symptoms (Gozlan et al., 2005; Kocan and Hershberger, 2006; Andreou et al., 2011, 2012). This is very well illustrated, for example, by the rosette agent *Sphareothecum destruens*, which has been rapidly spreading all over Europe via an invasive healthy fish host carrier (Gozlan et al., 2005; Gozlan, 2012). This fungal—like pathogen is intracellular, causing high mortality (up to 90%) after about 20–30 days but it can only be confidently detected by PCR analysis (Mendonca and Arkush, 2004). The paradox is that despite huge pan-continental population extinction, it remains difficult to characterize the true ecological impact of fungal and fungal-like pathogens on freshwater fish populations.

Despite fisheries representing a key ecosystem service for millions of people around the world, the full appreciation of disease risk associated with fungal pathogen emergence remains limited (Gozlan et al., 2006). Here, we review the current knowledge gaps of fungal microbes in fish, their phylogeography along with the current methods of detection and associated limitations and a global ecological understanding of their impacts on fish host populations. With an increasing volume of fish translocation around the world for farming and sport fishing, the relative absence of fish-infecting fungi outbreaks when compared to other more easily observed taxa exemplifies the current concern of a reporting bias in wild fish populations (Gozlan, 2012).

## Diversity and phylogeny

In recent years interest in the phylogeny of eukaryotes has been re-evaluated (see Adl et al., 2005). Based on data from sequencing genes, particularly rRNA gene sequences, Baldauf (2003) reassigned eukaryotes into eight different branches or supergroups within the tree of life, namely the opisthokonts, amoebozoa, plants, cercozoa, alveolates, heterokonts, discicristates, and excavates. The true fungi and Mesomycetozoea are placed along with the animals in the Opistokonta. The Mesomycetozoea form a clade, which falls on the animal branch, near the animal fungal divide (Paps et al., 2013). All of the Oomycota are placed into the Heterokont supergroup.

True fungi constitute the most species rich group of organisms on earth with 35 recognized classes and 129 orders (Hibbett et al., 2007). The majority of the fungi causing infection in fish belong to the phylum Ascomycota, with thick-walled non-motile spores (Hibbett et al., 2007). Within the Ascomycota, species from several genera have been reported to be associated with fish infections (Table 1). In addition to the Ascomycota, species belonging to the (earlier diverging) Zygomycota have also been reported to cause disease. The majority of the fungi, which can cause infection in fish are opportunistic and not exclusive parasites of fish. They are most commonly known as plant pathogens (e.g., *Penicillium corylophilum* and *Phoma herbarum*), soil fungi (e.g., *Paecilomyces lilacinus*) and some have even been reported to cause infection in immunosuppressed humans (e.g., *Exaphiala xenobiota* and *Ochroconis humicola*).

**Table 1:** **List of Fungi, Mesomycetozoea and Oomycetes species, which have been recorded as fish parasites in the Web of Knowledge since 1997**.

**Species**	**Order**	**Reported hosts**	**Generalist index**	**References**
**FUNGI**				
*Cladosporium sphaerospermum*	Capnodiales	*Lutjanus campechanu*	NA	Blaylock et al., 2001
*Exophiala angulospora*	Chaetothyriales	*Gadus morhua*	NA	Gjessing et al., 2011
*Exophiala pisciphila*	Chaetothyriales	*Stegostoma fasciatum*	NA	Marancik et al., 2011
*Exophiala xenobiotica*	Chaetothyriales	*Pseudocaranx dentex*	NA	Munchan et al., 2009
*Paecilomyces lilacinus*	Eurotiales	*Clarias gariepinus*	2	Rand et al., 2000a,b; Ali et al., 2011
		*Oreochromis niloticus niloticus*		
		*Tilapia aurea*		
*Penicillium corylophilum*	Eurotiales	*Lutjanus campechanus*	NA	Blaylock et al., 2001
*Ochroconis humicola*	Incertae sedis	*Pseudocaranx dentex*	3.3	Wada et al., 2005; Munchan et al., 2009
		*Pagrus major*		
		*Sebastiscus marmoratus*		
*Mucor circinelloides*	Mucorales	*Pelteobagrus fulvidraco*	5	Ke et al., 2010; Marancik et al., 2011
		*Pseudocaranx dentex*		
*Phoma herbarum*	Pleosporales	*Clarias gariepinus*	4	Faisal et al., 2007; Ali et al., 2011
		*Oncorhynchus tshawytscha*		
		*Oreochromis niloticus niloticus*		
*Phialemonium dimorphosporum*	Sordariales	*Mugil cephalus*	NA	Sosa et al., 2007a,b
*Ochroconis humicola*	Incertae sedis	*Pseudocaranx dentex*	3.3	Wada et al., 2005; Munchan et al., 2009
		*Pagrus major*		
		*Sebastiscus marmoratus*		
**MESOMYCETOZOEA**				
*Dermocystidium cyprini*	Dermocystida	*fluviatilis*	3.3	Lotman et al., 2000; Pekkarinen and Lotman, 2003
		*Gymnocephalus cernuus*		
		*Cyprinus carpio*		
*Dermocystidium fennicum*	Dermocystida	*PercaPerca fluviatilis*	NA	Pekkarinen and Lotman, 2003
*Dermocystidium koi*	Dermocystida	*Cyprinus carpio*	NA	Gjurcevic et al., 2008
*Dermocystidium percae*	Dermocystida	*Perca fluviatilis*	NA	Morley et al., 2008
*Dermocystidium branchiale*	Dermocystida	*Salvelinus alpinus*	2	Kristmundsson and Richter, 2009
		*Salmo trutta*		
*Sphaerothecum destruens*	Dermocystida	*Abramis brama*	3.6	Arkush et al., 1998; Gozlan et al., 2005; Andreou et al., 2012; Paley et al., 2012
		*Cyprinus carpio*		
		*Leucaspius delineatus*		
		*Oncorhynchus kisutch*		
		*Oncorhynchus mykiss*		
		*Oncorhynchus tshawytscha*		
		*Pseudorasbora parva*		
		*Rutilus rutilus*		
		*Salmo salar*		
		*Salmo trutta*		
		*Salvelinus fontinalis*		
*Ichthyophonus hoferi*	Ichthyophonida	*Citharichthys stigmaeus*	3.6	Rahimian, 1998; Criscione et al., 2002; Hershberger et al., 2002; Schmidt-Posthaus and Wahli, 2002; Gavryuseva, 2007; Kocan et al., 2010; Kramer-Schadt et al., 2010; Rasmussen et al., 2010; Gregg et al., 2012; Hamazaki et al., 2013
		*Clupea harengus*		
		*Clupea pallasi*		
		*Hypomesus pretiosus*		
		*Microgadus proximus*		
		*Oncorhynchus kisutch*		
		*Oncorhynchus mykiss*		
		*Oncorhynchus tshawytscha*		
		*Pleuronectes flesus*		
		*Salmo trutta*		
		*Sebastes alutus*		
		*Sebastes emphaeus*		
		*Sebastes flavidus*		
		*Sprattus sprattus*		
*Ichthyophonus irregularis*	Ichthyophonida	*Limanda ferruginea*	NA	Rand et al., 2000a,b
**OOMYCETES**				
*Achlya bisexualis*	Saprolegniales	*Mugil cephalus*	NA	Sosa et al., 2007a
*Achlya klebsiana*	Saprolegniales	*Oreochromis niloticus niloticus*	2.3	Ali et al., 2011; Cao et al., 2013
		*Clarias gariepinus*		
		*Pelteobagrus fuvidraco*		
*Achlya americana*	Saprolegniales	*Coregonus lavaretus holsatus*	NA	Czeczuga et al., 2004
*Achlya. oblongata*	Saprolegniales	*Coregonus lavaretus holsatus*	NA	Czeczuga et al., 2004
*Achlya racemosa*	Saprolegniales	*Odonthestes bonariensis*	NA	Pacheco Marino et al., 2009
*Achlya ambisexualis*	Saprolegniales	*Oncorhynchus mykiss*	NA	Vega-Ramirez et al., 2013
*Aphanomyces parasiticus*	Saprolegniales	*Coregonus lavaretus holsatus*	NA	Czeczuga et al., 2004
*Aphanomyces frigidophilus*	Saprolegniales	*Coregonus lavaretus holsatus*	2	Czeczuga et al., 2004, 2005
		*Salmo trutta*		
*Aphanomyces invadans*	Saprolegniales	*Alosa sapidissima*		
		*Anguilla anguilla*		
		*Ameiurus melas*		
		*Archosargus probatocephalus*		
		*Bairdiella chrysoura*		
		*Brevoortia tyrannus*		
		*Brycinus lateralis*		
		*Barbus poechii*		
		*Barbus paludinosus*		
		*Barbus unitaeniatus*		
		*Catla catla*		
		*Channa marulius*		
		*Clarias gariepinus*		
		*Clarias ngamensis*		
		*Cyprinus carpio*		
		*Fundulus heteroclitus*		
		*Fundulus majalis*		
		*Hepsetus odoe*		
		*Hydrocynus vittatus*		
		*Ictalurus punctatus*		
		*Leiopotherapon unicolor*		
		*Labeo lunatus*		
		*Labeo cylindricus*		
		*Lepomis macrochirus*		
		*Macquaria ambigua*		
		*Maccullochella peelii*		
		*Marcusenius macrolepidotus*		
		*Micralestes acutidens*		
		*Micropterus salmoides*		
		*Mugil cephalus*		
		*Mugil curema*		
		*Nematalosa erebi*		
		*Oncorhynchus mykiss*		
		*Oreochromis andersonii*		
		*Oreochromis macrochir*		
		*Petrocephalus catostoma*		
		*Pharyngochromis acuticeps*		
		*Pogonias cromis*		
		*Sargochromis codringtonii*		
		*Sargochromis giardi*		
		*Serranochromis robustus*		
		*Serranochromis angusticeps*		
		*Serranochromis macrocephalus*		
		*Schilbe intermedius*		
		*Silurus glanis*		
		*Tilapia sparrmanii*		
*Tilapia rendalli*		*Trinectus maculates*	3.7	Thompson et al., 1999; Hawke et al., 2003; Harikrishnan et al., 2005; Kiryu et al., 2005; Webb et al., 2005; Vandersea et al., 2006; Sosa et al., 2007b; Oidtmann et al., 2008; Saylor et al., 2010; Boys et al., 2012; Go et al., 2012; Huchzermeyer and Van der Waal, 2012; Saikia and Kamilya, 2012
*Aphanomyces irregularis*	Saprolegniales	*Coregonus lavaretus holsatus*	NA	Czeczuga et al., 2004
*Aphanomyces laevis*	Saprolegniales	*Aplocheilus panchax*	4	Mondal and De, 2002; Ali et al., 2011
		*Clarias gariepinus*		
		*Oreochromis niloticus niloticus*		
*Aphanomyces salsuginosus*	Saprolegniales	*Salangichthys microdon*	NA	Takuma et al., 2010
*Saprolegnia australis*	Saprolegniales	*Oncorhynchus nerka*	3.3	Hussein et al., 2001; Chang et al., 2002; Fregeneda-Grandes et al., 2007
		*Plecoglossus altivelis*		
		*Salmo trutta*		
*Saprolegnia brachydanis*	Saprolegniales	*Danio rerio*	NA	Ke et al., 2009a,b
*Saprolegnia diclina*	Saprolegniales	*Acipencer persicus*	3.3	Leano et al., 1999; Fregeneda-Grandes et al., 2007; Ghiasi et al., 2010; Shahbazian et al., 2010; Thoen et al., 2011
		*Oncorhynchus mykiss Salmo salar* eggs		
		*Salmo trutta*		
		*Sciaenops ocellatus*		
*Saprolegnia ferax*	Saprolegniales	*Carassiuus auratus*	3.6	Czeczuga et al., 2004; Fregeneda-Grandes et al., 2007; Ke et al., 2009a,b; Pacheco Marino et al., 2009; Shahbazian et al., 2010; Cao et al., 2013
		*Coregonus lavaretus holsatus*		
		*Odonthestes bonariensis*		
		*Oncorhynchus mykiss* eggs		
		*Salmo trutta*		
*Saprolegnia furcata*	Saprolegniales	*Salmo trutta*	NA	Fregeneda-Grandes et al., 2007
*Saprolegnia hypogyana*		*Oncorhynchus mykiss* eggs	2	Fregeneda-Grandes et al., 2007; Shahbazian et al., 2010
		*Salmo trutta*		
*Saprolegnia parasitica*	Saprolegniales	*Acipencer persicus*		
		*Astyanax eigenmanniorum*		
		*Astyanax fasciatus*		
		*Bidyanus bidyanus*		
		*Coregonus lavaretus holsatus*		
		*Ictalurus punctatus*		
		*Odontesthes bonariensis*		
		*Oncorhynchus mykiss*		
		*Oncorhynchus masu*		
		eggs *Oncorhynchus nerka*	3.3	Bangyeekhun et al., 2001; Hussein and Hatai, 2002; Czeczuga et al., 2004; Fregeneda-Grandes et al., 2007; Mancini et al., 2008, 2010; Mifsud and Rowland, 2008; Ghiasi et al., 2010; Shahbazian et al., 2010; Thoen et al., 2011
		*Salmo salar* eggs *Salmo trutta*		
		*Salvelinus leucomaenis*		
*Saprolegnia polymorpha*	Saprolegniales	*Cyprinus carpio*	NA	Willoughby, 1998
*Saprolegnia salmonis*	Saprolegniales	*Coregonus lavaretus holsatus*	2.4	Hussein et al., 2001; Chang et al., 2002; Hussein and Hatai, 2002; Czeczuga et al., 2004, 2005
		*Oncorhynchus masu*		
		*Oncorhynchus mykiss*		
		*Oncorhynchus nerka*		
		*Plecoglossus altivelis*		
		*Salmo trutta*		
		*Salvelinus leucomaenis*		
*Saprolegnia shikotsuensis*	Saprolegniales	*Coregonus lavaretus holsatus*	NA	Czeczuga et al., 2005
*Pythium aquatile*	Pythiales	*Coregonus lavaretus holsatus*	NA	Czeczuga et al., 2004
*Pythium pulchrum*	Pythiales	*Coregonus lavaretus holsatus*	NA	Czeczuga et al., 2004
*Pythium thalassium*	Pythiales	*Coregonus lavaretus holsatus*	NA	Czeczuga et al., 2004
*Pythium torulosum*	Pythiales	*Coregonus lavaretus holsatus*	NA	Czeczuga et al., 2004

A generalist index was calculated for each parasite using the method described in Poulin and Mouillot (2003); where species with two or more hosts can have generalist indices ranging from 1 (all host species share the same genus) to 5, using the five taxonomic levels of genus, family, order, class, and phylum. NA stands for non-applicable as the index cannot be calculated when only one host has been reported. The fish taxonomy proposed by Nelson (1994) was used in calculating all generalist indices.

Branching close to the divergence between fungi and animals there is a relatively recently recognized clade of organisms, the Mesomycetozoea (Mendoza et al., 2002; Ragan et al., 1996), which includes a number of species that are pathogenic to aquatic organisms including fish (Mendoza et al., 2002; Glockling et al., 2013). Within the Mesomycetozoea, species can be divided further into the orders of Dermocystida and Ichthyophonida. The Dermocystida include a number of species that can be pathogenic to fish, the most notable being *Sphaerothecum destruens,* which can infect a wide range of hosts and has been shown to cause disease and high mortality in cyprinids (Andreou et al., 2011, 2012) and salmonid species (Arkush et al., 1998; Paley et al., 2012). The order also includes numerous *Dermocystidium* sp., which can infect a variety of fish species (see Table 1). The diversity of the *Dermocystidium* genus is probably underestimated as a large proportion of recorded cases in the literature only identify the pathogen to genus level. This can be addressed by applying molecular techniques to identify species. Within the Ichthyophonida, *Ichthyophonus hoferi* is the most common parasite of salt and freshwater fish (Hershberger et al., 2010; Kocan et al., 2010; Gregg et al., 2012; Hamazaki et al., 2013).

The Oomycete parasites of fishes are placed in the Phylum Oomycota and fall into either the saprolegnialean lineage or the peronosporalean lineage. The Oomycetes are water moulds which morphologically resemble fungi, but are taxonomically distinct, encompassing species that are parasitic to a large diversity of host species (Beakes et al., 2012). The majority of the species, which can infect and cause disease in fish belong to the order of Saprolegniales and fall within the genera of *Saprolegnia*, *Aphanomyces* and *Achlya*. A smaller number of species fall within the genus *Pythium*, a member of the peronsporalean lineage. Twelve species of *Saprolegnia* and six species each of *Aphanomyces* and *Achlya* (Table 1) are more often described in the literature as causing infection in fish; with the most common pathogens of fish being *Saprolegnia parasitica* and *Aphanomyces invadans* which have relatively high generalist indices (See Table 1). *S. parasitica* has been reported to cause disease in 12 fish species whilst *A. invadans* can parasitize 48 fish species.

## Host specificity

A common characteristic of the fish pathogens within Fungi, Mesomycetozoea, and Oomycetes is their generalist nature, with the majority of species infecting and causing disease in fishes across different families (Table 1). All three groups include an equal proportion of species with generalist indices above 3 indicating that they are true generalists (Poulin and Mouillot, 2003). Due to higher reporting and detection of disease in farmed environments, most disease reports are from aquaculture facilities and involve cultured fish species. There is thus a bias in the fish species reported as susceptible to these pathogens and a possible underestimation of their generalist nature (Ramaiah, 2006). A large number of species have a single record of affecting a single fish species in the literature and thus the generalist index cannot be calculated.

The ability of fungal and fungal-like pathogens to infect multiple hosts (“the widest spectrum of host ranges for any group of pathogens” according to Fisher et al., 2012; see Table 1 for fish). often drives high virulence in the most susceptible hosts (Andreou et al., 2012; Huchzermeyer and Van der Waal, 2012). The aspect of generalism in pathogenicity is important due to the fact that generalist pathogens are more likely to emerge through host switching (Woolhouse and Gowtage-Sequeria, 2005), and it is often overlooked (Yamamoto and Kilistoff, 1979; Peeler et al., 2010). However, it is commonly accepted (Ewald, 1994) that in single hosts the optimum level of virulence is determined by the trade-off between virulence and transmissibility (Davies et al., 2001). Thus, the composition of the community and the susceptibility of each host could alter its potential transmissibility and the outcome of infection (Woolhouse et al., 2001). Experimental challenges to fungal and fungal-like pathogens of several fish host species are currently needed. This could involve simple one host–one pathogen challenges such as in Andreou et al. (2012) or a combination of multi-hosts challenges. In addition, experimental data on the free-living elements of these life cycles of pathogens such as the presence of zoospores, would allow the measurement of their production, longevity in the system and their resistance to a range of abiotic factors (e.g., temperature, PH). These data are needed to build reliable models to test host susceptibility, understand the controlling factors of infectious phase as well as the recovery phase typical of SIT or SEIR epidemiological models (susceptible-exposed-infectious-recovered).

## Life cycles and stages

In the assimilative phases of oomycetes and most of the true fungi, colonization of new tissues is accomplished through the growth of hyphae, with the exception of the black yeasts, *Exophiala*, which may transition between yeast and hyphal forms (dimorphism) (de Hoog et al., 2011). Mesomycetozoeans more often grow as round multinucleate coenocytes. These can be concentrated in visible cysts in the genus *Dermocystidium* (e.g., Lotman et al., 2000) or disseminated or nodular in *S. destruens* and *Ichthyophonus* (Sindermann and Scattergood, 1954; Arkush et al., 1998). Hyphal forms have been described in some *Dermocystidium* (Dykova and Lom, 1992) and are common in *Ichthyophonus* (Sindermann and Scattergood, 1954; Rand, 1994; Franco-Sierra and Alvarez-Pellitero, 1999). Only true fungi, however, have septate hyphae, although some oomycetes have segmented or plugged thalli and thus are also compartmentalized.

All fungal and fungal-like pathogens have prolific asexual reproduction (r-strategy) functioning for dispersal or further dissemination within the host. In the fungi, this is through the production of conidiospores (Ascomycota) or sporangiospores (Zygomycota), and the budding of yeast stages. These spores are not motile and are protected by a chitinous cell wall. The durability and resilience of these spores is an important adaptation for increasing opportunities to encounter new susceptible hosts (Fisher et al., 2012). These spores can survive in a dormant state during conditions unfavorable for growth. Oomycetes produce biflagellated zoospores within sporangia, usually located either at the terminal ends of hyphae. These spores function to disperse the parasite between hosts and typically encyst after a short period of motility. In *Saprolegnia* species longer lived secondary zoospores emerge from cysts produced by primary zoospores. This pattern of re-emergence called polyplanetism, may be repeated several times (Bruno et al., 2011), and most likely functions to allow several opportunities to contact a new host. Zoospores of many oomycetes are chemotactic, responding to amino acids, carbohydrates and a range of aldehyde attractants (Donaldson and Deacon, 1993). The encysted zoospores of *S. parasitica* are decorated by long hooked hairs that are thought to aid in attachment to the fish host (Van West, 2006; Walker and Van West, 2007).

Reproduction in the Mesomycetozoea is more varied (Mendoza et al., 2002). *S. destruens* produce non-motile walled endospores which may either infect other cells within the same host or spread and infect a new host (Arkush et al., 2003). Endospores also produce singly flagellate zoospores upon exposure to fresh water (Arkush et al., 2003) but it is not clear whether these zoospores are infective (Paley et al., 2012). *Dermocystidium* has similar development with zoospore development within spores, but zoospores are infective (Olson et al., 1991). Released endospores of both *Dermocystidium salmonis* and *S. destruens* have the capacity to release zoospores for several weeks at 4°C (Olson et al., 1991; Andreou et al., 2009). The life cycle of *Ichthyophonus* is less understood and varies with pH (Okamoto et al., 1985; Spanggaard et al., 1995; Franco-Sierra and Alvarez-Pellitero, 1999). Single and multinucleate endospores are produced in culture and *in vivo* (Okamoto et al., 1985; Spanggaard et al., 1995; Franco-Sierra and Alvarez-Pellitero, 1999). Motile zoospores are not produced but amoeboid stages are released under specific pH optima in culture (e.g., Okamoto et al., 1985). Transmission is also not well understood, except that the parasite can be acquired through carnivory (Jones and Dawe, 2002). Kocan et al. (2013) describe small amoeboid stages within the stomach wall of sculpin and trout hosts after feeding of infected tissues and hypothesize that these amoebae represent the infectious stage. The infectious stage of planktivorous fish is still unknown (Gregg et al., 2012) and an alternate host is suspected (Sindermann and Scattergood, 1954).

In parasites of fishes sexual reproduction (s-strategy) has only been described in a few oomycetes. When sexual reproduction occurs, the two dissimilar gametangial structures called the oogonium and the antheridium grow closer together until they fuse, and haploid nuclei from the antheridia fertilize the eggs within the oogonia forming diploid oospores. In free living oomycetes the fertilized zygote, or oospore, is typically resistant and can survive for prolonged periods. Meiosis and recombination occur before germination of the oospore. However, the main oomycete pathogens of live fish (e.g., *A. invadans* and *S. parasitica*), do not generally (in case of *A. invadans* never) reproduce sexually and therefore rely entirely on asexual zoosporogenesis (r-strategy). Some egg infecting species do produce oogonia (e.g., *S. australis*, *S. diclina*, *S. ferax*) but even in these species oospore germination is rarely if ever observed. It is highly unlikely that oospores serve as effective resistant survival structures for fish parasitic oomycetes. Most true fungal parasites of fish are described as “fungi imperfecti,” based on the lack of a described sexual stage.

## Trophic modes

Research on animal parasites has revealed that many of these species are not exclusively saprophytic or parasitic (Gleason et al., 2010; McCreadie et al., 2011). In fact, their precise ecological functions can only be understood with intensive metagenomic investigations, which have rarely been conducted (Jiang et al., 2013). Nonetheless, these microorganisms are frequently characterized as either saprotrophs or biotrophs (Gleason et al., 2010; McCreadie et al., 2011). Saprotrophs usually do not infect live hosts, rather they grow on non-living organic material. In contrast many biotrophs cannot grow outside the host, but some can be grown in culture. Growth of parasites in culture allows research on mechanisms of infection and sequencing genes. Facultative parasites can grow well as either parasites or saprotrophs. Many eukaryotic microorganisms are thought to be parasites primarily because they cannot be grown outside their host, but in fact their trophic relationships remain to be determined. Many Oomycete species are primarily saprotrophs, yet few can become parasites under certain conditions, such as compromised immunity in their hosts. The important point is that they have alternative substrates for growth outside the host, which is an important characteristic of emerging infectious diseases (EID) (Fisher et al., 2012).

## Proteins as substrates for growth

For a long time proteins have been known to be good substrates for the isolation of Oomycetes into pure culture and for their subsequent growth in liquid media (Sparrow, 1960). For example, casein and keratin can be useful substrates for isolation and growth. Furthermore animal hosts and tissues are known to be protein rich environments. Czeczuga et al. (2002) isolated many species of Oomycetes from specimens of fish muscles placed in freshwater lakes. Some of these specimens came from fish, which were known to be hosts for Oomycetes. Smith et al. (1994) demonstrated proteolytic activity of *Saprolegnia diclina, ferax,* and *parasitica* by observing the clearing of casein on solid media. Proteins must be digested extra-cellularly and the amino acids produced must be transported into the cell prior to their catabolism. Jiang et al. (2013) documented the presence of genes for serine, metallo- and cysteine proteases and genes for amino acid transporters in the complete sequence of the genome of *S. parasitica*.

Saprotrophic isolates of *Saprolegnia*, *Achlya*, *Dictyuchus*, *Leptolegnia*, *Aphanomyces*, *Apodachlya,* and *Pythium* grew rapidly on many but not all amino acids as sole sources of carbon and nitrogen in liquid media (Gleason et al., 1970a,b; Faro, 1971). Alanine, proline, glutamate, aspartate, leucine, lysine, arginine, serine, and phenylalanine were especially good carbon sources, there was very little or no growth on valine, isoleucine, threonine, methionine, and glycine, and there were considerable differences in rates of utilization among the species tested. Saprotrohic and parasitic isolates of *Saprolegnia* can remove all amino acids from liquid media during growth on mixtures of amino acids (Gleason, 1973; Nolan, 1976). These data indicate that many Oomycetes have the capacity for digestion of proteins and subsequent uptake and catabolism of amino acids. Therefore they commonly grow in protein rich environments. Recently, a few species in the Mesomycetozoea have been grown in culture (Glockling et al., 2013), but nutritional experiments have not been conducted, and little is known about their proteolytic capacities.

## Current detection techniques

Lesions formed by parasites were initially characterized from phenotypic, serological and morphological properties of the pathogen. Isolation and culturing of causative organisms from swabbed lesions of infected fish has been an integral part in understanding the taxonomic groupings, etiology of the disease, infectivity, and host-parasite relationships. The process of isolating and identifying pathogens can however be a time consuming process requiring a high level of technical expertise.

Morphological identification of microbial species, which often requires identification of reproductive stages, is difficult to accomplish directly from ulcerated tissue. Direct visualization of pathogens in infected tissues has been made possible with the development of species-specific fluorescent probes. For example, the monoclonal antibody MAb 3gJC9, which is specific for an antigen involved in the pathogenicity of *Aphanomyces astaci* and *A. invadans (=piscicida)* in infected crayfish and fish respectively, has been used for immunofluorescent identification of these species in infected tissues (Miles et al., 2003). The approach was found to be more sensitive than the conventional staining method, Grocott's methanamine silver stain, in that it enabled the detection of early stages of infection (Grocott, 1955; Miles et al., 2003). Fluorescent hybridization (FISH) probes have also been used to identify specific pathogens in infected tissues *in situ*. For example, *A. invadans* was found to be a primary oomycete pathogen in ulcerative mycosis of infected estuarine fish in North Carolina and Florida using a FISH assay (Vandersea et al., 2006; Sosa et al., 2007a,b). Continuing improvements in isolation, culturing and *in situ* approaches is essential for broadening our understanding of disease pathology and etiology and more fundamentally the morphology and physiology of these pathogenic species. In comparison to morphological and physiological classification, the rapid advances in molecular techniques has improved the reliability and accuracy of the tool in distinguishing many taxa, such as the microsporidian taxa (Larsson, 2005). A molecular approach has also led to rapid development of diagnostic tools which involve polymerase chain reaction (PCR), amplification of nucleic acids, restriction enzyme digestion, probe hybridization and nucleotide sequencing. The development of the FISH assay for example was as a result of using a sensitive PCR technique. The use of PCR to detect and identify infections has become commonplace (Tsui et al., 2011). A large number of disease-causing pathogens are often identified to genus level (e.g., *Dermocystidium* sp.) and not species level. The number of species being identified has been constantly increasing through the use of molecular tools for disease detection and identification. A concerted effort to use the same DNA loci would increase the available genetic information resulting in a better resolution of the phylogenetic relationships within and between these groups. The 18S rRNA gene has been used extensively (for Fungi and Mesomycetozoans); however the Internal Transcribed Sequence 1 (ITS1) has been more extensively used within the Oomycetes. As documented by Diéguez-Uribeondo et al. (2007) for the *S. diclina- S. parasitica* complex, both molecular and morphological and physiological data can help solve phylogenetic relationships. Thus, using ITS rRNA gene, five phylogenetic separate clades were identified for the *Saprolegnia* complex, with all isolates collected from salmonid lesions falling into a single clade (i.e., clade I). However, within that clade I, parasitic isolates came from a wide range of hosts including, for example, crustaceans, and catfish but also non-pathogenic isolates from soil and water. Molecular analyses have the potential to discriminate at the subspecies or strain level (Phadee et al., 2004). The level of sensitivity of the molecular techniques in the clinical context has however been sporadic (Cunningham, 2002). This is largely due to the relatively low genomic information that is available through public data bases such as Genbank. We propose that all reported cases of disease outbreaks should have both of these regions sequenced and reported within the literature. The use of these loci will allow both detection at species level (18S rRNA) and identification of different strains (using ITS1) within the same species; allowing for a better identification and detection of virulent strains. This collection of information alongside morphological and physiological data will increase the resolution of the phylogenetic information and the sensitivity of molecular identification.

Alternative detection approaches include loop-mediated isothermal amplification (LAMP) and pyrolysis mass spectrometry. LAMP has the potential of increasing sensitivity of pathogen compared to PCR and unlike PCR it is not inactivated by tissue and blood-derived inhibitors or genomic DNA (Savan et al., 2005). It has been used in the detection of trypanosome infection (Savan et al., 2005). Pyrolysis mass spectrometry profile and canonical variate analysis have been used to demonstrate clusters of *A. invadans* isolates and discriminate them from non-pathogenic *Aphanomyces* species (Lilley et al., 2001). The development and refinement of multiple approaches of detection have their place in increasing the knowledge of the pathogen, its distribution, impact and possible management.

## Importance of fungal pathogens in aquaculture

Fish represent a key ecosystem service for fisheries and aquaculture across the world (Zhao et al., 2014). The annual harvest is about 42 million tonnes (marine and freshwater) and the sector employs 33.1 million people, highlighting the tremendous social cost of fisheries (Gozlan and Britton, 2014). The livelihoods of 60 million people in the developing world are dependent on river fisheries and millions more rely on them for food (Dugan and Allison, 2010). However, disease in aquaculture represents the most significant economic losses and in particular fungal infections, which in terms of economic impact are second only to bacterial diseases (Neish and Hughes, 1980; Noga, 1993; Bruno et al., 2011; Ramaiah, 2006; Van West, 2006; Gonçalves and Gagnon, 2011). For example it has been reported in Japan some annual losses of 50% in the production of coho salmon *Oncorhynchus kisutch* and elvers of eel *Anguilla Anguilla* due to outbreaks of *S. parasitica* (Hatai and Hoshai, 1994; Scarfe, 2003).

In the last decades, the aquaculture sector has seen a change in the fish production with a trend toward intensification with the use of recirculating systems (Larkin and Sylvia, 1999). The underpinning drive was a reduction of environmental footprint, a better control of the rearing environment and increased biosecurity. Nonetheless, this improved control of rearing conditions, has lead the industry to also increase the stocking densities of target fish. Thus, it has resulted in an increase of disease outbreaks, with faster transmission and increased mortalities (Bondad-Reantaso et al., 2005; Whittington and Chong, 2007; Peeler et al., 2010; Gonçalves and Gagnon, 2011).

One of the key risks associated with this new aquaculture environment is the stress caused by intensive production. Some fungal pathogens such as *Saproglegnia* for example are more prevalent and virulent in host (salmonids in particular) that are raised under stressful conditions (Willoughby and Pickering, 1977; Willoughby, 1978; Jeney and Jeney, 1995). However, other significant pathogen risks in aquaculture arise from the large and frequent movement of young stages due to either a lack of or insufficient national production, or due to fish species for which the life cycle has not yet been mastered at a commercial level, or even contaminated sources of water supplies. Of course this is not specific to fungal or fungal-like pathogens but their generalist and opportunistic nature associated with a wide environmental tolerance are risk factors that may lead to significant loss of production (Harrell et al., 1986; Paley et al., 2012).

In addition, mycoses spread in fishes are often seen as a secondary phenomenon. However, due to their virulence, their current emergence in wild fish populations and also the risk of spill back from aquaculture facilities to the wild, routine pathological examination should include (in addition to bacteriological ones) mycological examination (see Rehulka, 1991 for details). Dominant fungal pathogens reported in aquaculture are oomycetes including the genera *Achlya*, *Aphanomyces* and *Saprolegnia* (Willoughby and Pickering, 1977; Blazer and Wolke, 1979; Noga, 1993). *A. invadans* for example can cause epizootic ulcerative syndrome in over a hundred of mostly freshwater fish (e.g., Vishwanath et al., 1998; Blazer et al., 2013; Nsonga et al., 2013) but also in some brackish fish species (Catap and Munday, 2002; Sosa et al., 2007b). In aquaculture conditions, the most appropriate control is through eradication of the stock, quarantine of new stocks and good husbandry (Scarfe, 2003; Whittington and Chong, 2007) and as such represent a significant cost to the trade (Forneris et al., 2003).

There are no treatments that are specific to fungal and fungal-like pathogens but existing ones such as the use of hydrogen peroxide or formalin (Arndt et al., 2001), malachite green (Van West, 2006), sodium chloride (Schreier et al., 1996) and bronopol (Shinn et al., 2012) all present some significant issues related to either human or fish health or to efficacy of the treatments (Carana et al., 2012). Malachite green was banned by the US and EU in early nineties and since then formalin has probably been the most effective control measure but there is a strong possibility that this will soon also be banned from use. Other treatment such as bronopol and other agents are not as effective. Other treatments such as the use of ozone in recirculating systems have to be specifically adapted for fugal pathogens. For example, studies have shown that ozone treatments for *Saprolegnia* are effective with doze from about 0.01 to 0.2 mg.L^−1^ (Gonçalves and Gagnon, 2011) but present a cost of through reduction in hatching rates (e.g., 42.6–49.1%). New treatments based on plant extract have shown some promising paths but further evaluations need to be performed before its use by the industry (Carana et al., 2012).

## Ecological impact on wild populations

The emergence of infectious diseases caused by fungal and fungal-like microbes continues to negatively impact wild fish populations, leading in some cases to local and pan-continental extinctions (Gozlan et al., 2005, 2010; Rowley et al., 2013). Thus, understanding of the true ecological cost of fungal and fungal-like microbes is pivotal to improve our conservation practices of fish populations, especially freshwater species, as declines in populations, species distributions and species diversity continue to occur at alarming rates (Myers, 1993; Singh, 2002; Romansic et al., 2009).

Fungal and fungal-like microbes that cause disease emergence in wild fish (Table 2), crayfish, amphibians and other aqutic taxa include *Saprolegnia, Batrachochytrium, Ichthyophonous, Aphanomyces, Achyla* and *Sphaerothecum* (Bruno et al., 2011; Swei et al., 2011). *Saprolegnia* and *Sphaerothecum* spp. are impacting wild salmon populations around the world (Willoughby et al., 1983; Van West, 2006; Andreou et al., 2009), prevailing in 32% of adult late-fall-run chinook salmon returning to Battle Creek on the Upper Sacramento River (Arkush et al., 1998). *Aphanomyces* spp. are also responsible for causing EIDs, for example, Epizootic Ulcerative Syndrome (EUS) commonly known as red spot in over a hundred freshwater and estuarine fish species worldwide (Chinabut et al., 1995; Lilley et al., 1997; Boys et al., 2012). EUS has been recognized in Australia (Huchzermeyer and Van der Waal, 2012) and the Philippines (Callinan et al., 1995) since 1972 and 1995 respectively, however in 2006 this fungal pathogen was sighted in the Zambezi River System (ZRS), Africa, the pathogen had travelled further along the ZRS inhabiting several new ecosystems (Huchzermeyer and Van der Waal, 2012; Nsonga et al., 2013). Additionally, *Aphanomcyes* spp. low host specificity increases its prevalence among a range of species, increasing disease outbreaks in the ZRS, which is home to approximately eighty species and thus becoming a great concern in disease control (Huchzermeyer and Van der Waal, 2012).

**Table 2:** **Example of fungal infections in wild fish population**.

**Host (Family)**	**Pathogen**	**Location**	**Prevalence**	**Mortality**	**References**
**SALMONIDS**					
*Oncorhynchus tshawytscha* (Chinook Salmon)	*Saprolegnia parasitica*	Columbian & Snake Rivers,	–	22%	Neitzel et al., 2004
*Oncorhynchus mykiss* (Rainbow Trout)		United States of America.			
*Salmo salar* (Atlantic Salmon)	*Saprolegnia diclina*	River North Esk, Scotland.	30%	–	Roberts et al., 1972
*Salmo trutta* (Sea Trout)					
*Oncorhynchus tshawytscha*	*Sphaerothecum destruens*	Sacramento River, United States of America.	32%	–	Arkush et al., 1998
**CLUPEDIDS**					
*Clupea harengus* (Bony Herring)	*Ichthyophonus hoferi*	Skagerrak-Kattegat Area, Sweden.	1.1%	8.9%	Rahimian and Thulin, 1996
**CHARACIDS**					
*Astyanax eigenmanniorum*	*Saprolegnia parasitica*	Central Argentina.	95%	–	Mancini et al., 2008
*Astyanax fasciatus*					
**CICHLIDS**					
*Sargochromis giardia* (Pink Bream)	*Aphanomyces invadans* (EUS)	Zambezi River System, Africa.	3–37.5%	–	Huchzermeyer and Van der Waal, 2012; Nsonga et al., 2013
*Brycinus lateralis* (Stripped Robber)					
**CYPRINIDS**					
*Pseudorasbora parva* (Topmouth Gudgeon)	*Sphaerothecum destruens*	Meuse River, Netherlands.	67–74%	–	Spikmans et al., 2013
*Leucaspius delineates* (Belica)	*Sphaerothecum destruens*	Stoneham Lakes system, United Kingdom.	5%	–	Andreou et al., 2011
**PERCIDIDS**					
*Leiopotherapon unicolor* (Spangled Perch)	*Aphanomyces invadans* (EUS)	Murray-Darling River System, Australia.	10% (2008)	–	Boys et al., 2012
*Macquaria ambigua* (Golden Perch)			29% (2010)		

However, compared to farmed fish populations, monitoring EIDs in wild populations can prove difficult as fish are constantly moving long distances beneath turbid waters, which means they can go undetected and underreported distorting our understanding of the effects on these populations (Gozlan, 2012). Globally, EIDs have caused high mortalities in farmed populations (Torto-Alalibo et al., 2005; Phillips et al., 2008; Van Den Berg et al., 2013). This is important information for wild populations because there are several instances where transmission of fungal and fungal-like microbes can occur between the two environments. For example, farmed fisheries often drain into rivers (Andreou et al., 2012) allowing the transfer of microbes and other organisms (Krkosek et al., 2005; Hilborn, 2006). *Pseudorasbora parva* (topmouth gudgeon), widely known by its aquaculture and ornamental fish trade, is a healthy carrier of *S. destruens* (Gozlan et al., 2005). Originally and unintentionally imported from China, the topmouth gudgeon's propitious nature has allowed it to become a profound invader in wild environments, invading thirty five new countries over the past 40 years, where for example *S. destruens* was identified in river systems of the Netherlands and the UK, posing great threats to native fish populations (Gozlan et al., 2010; Spikmans et al., 2013).

Previous studies have shown that susceptibility of farmed fish to fungal or fungal-like microbes depends on several factors including rapid drops in ambient temperatures (Bly et al., 1993; Lategan et al., 2004), low water levels, failure to remove dead fish or eggs, primary infection by other organisms, (Piper et al., 1982; Plumb, 1984) and pollution (Wu et al., 2010), all of which can reduce ecosystem function (Chapin et al., 2000; Cowx and Gerdeaux, 2004; Peeler et al., 2010) and lead to an increase in EIDs (Woolhouse and Gowtage-Sequeria, 2005). Thus, we could expect that such environmental drivers at play in the wild would potentially have also a direct impact on the emergence of fungal pathogens. In particular, the recent paper by Vörösmarty et al. (2010) shows that 65% of rivers worldwide regarding thermal and water level disturbances are under moderate-high threat, in particular Asia and North America. However, Vörösmarty et al. (2010) goes on further to highlight the fact that there is a lack of knowledge and investment being directed to biodiversity conservation, with an increase in EIDs, species distinctions, human population, climate change and habitat destruction (Vörösmarty et al., 2010; Huchzermeyer and Van der Waal, 2012; Nsonga et al., 2013). It will be important to monitor these river systems and to reduce these pressures, consequently allowing the populations, species distributions and diversity of fish to remain sustainable for the future.

## Perspectives and conclusions

Since, the initial discovery of the fungal chytrid pathogen 20 years ago (Berger et al., 1998), several studies have reported its significant impact on amphibians along with major population declines worldwide (Skerratt et al., 2007). What is interesting with this particular pathogen is the relatively good epidemiological data, which have allowed the progression of the disease to be tracked on a global scale and in many wild amphibian populations.

However, there is currently not enough epidemiological data related to fungal pathogens of fish. In light of the recent emergence of *S. destruens*, which poses a threat to European fish diversity (Gozlan et al., 2005; Andreou et al., 2012), it is likely that patterns of ecological impacts similar to those found in chytrid parasites of amphibians, are at play in freshwater fish populations (Gozlan, 2012). For example, as the great majority of *S. destruens* cases are driven by the invasion of a healthy fish carrier, it is expected, as shown by Spikmans et al. (2013), that additional monitoring of invaded wild fish communities would show the presence of this fungal-like infectious pathogen. In fact, there is not enough fungal pathogen data mostly for fish from wild populations. It is interesting to note that the dominant reporting of fish fungal and fungal-like pathogens has come from the aquaculture sector with very limited reports on fungal emergence in wild fish populations (see Table 2). The key reason is likely to be a combination of a lack of external pathological specificity of infected hosts and the chronic nature of some of the diseases caused by these fungal and fungal-like pathogens, which, in contrast to viral pathogens, spread over longer periods of time.

Research should be modeled after the amphibian chytrid research structure with a lot more systematic tracking of these pathogens. For example, the recent paper by McMahon et al. (2013) clearly indicates that even for the chytrid, which has been well studied in the wild, new potential non-amphibian hosts could contribute further to its dispersal, prevalence and virulence. Similarly, it would thus be pertinent to determine if fungal and fungal-like pathogens of fish that have a high generalist index could include non-fish hosts and thus contribute to a wider dissemination of some fungal related diseases beyond the immediate local fish communities. Additional monitoring of wild fishes is also needed. Identification and surveys of environmental drivers of fungal pathogens would improve understanding of the ecological risks of disease emergence in aquatic communities (Copp et al., 2009, 2010). This should also be the concern of the aquaculture sector, as strong pathways exist between wild and farmed fish with truly biosecure fish farms being the exception. The pan-extinction of sunbleak *Leucaspius delineatus* populations in Europe in less than 40 years should be a reminder of the risk associated with an un-controlled epizooty of fungal pathogens (see Gozlan et al., 2005, 2010). Environmental surveys to identify ecological drivers of fungal pathogens in fishes are also key in characterizing the underpinning drivers of fungal and fungal-like pathogen emergence. For example, fish fungal pathogen emergence such as the EUS in Africa could well be linked to current environmental changes occurring in African rivers (Vörösmarty et al., 2010). Thus, in light of the importance of freshwater fish for millions of people around the world, particularly in developing countries, in addition to biodiversity conservation perspectives, pathologists should make a concerted effort to increase their monitoring of fungal pathogens in wild fish populations. Currently, PCR is the method that should be used for monitoring. Along with an increasing reduction in PCR associated cost, their sensitivity and specificity should facilitate such regular monitoring of the wild fish compartment.

In conclusion, our review of fungal and fungal-like pathogens of fish has highlighted current knowledge gaps that need to be rapidly filled if future epizootics are to be prevented. It has also indicated that epidemiological elements arising from other non-fish specific fungal pathogens could be used to refine our true understanding of current and future ecological impacts of these types of pathogens on global fish diversity. For example, existing experimental data arising from fungal pathogen challenges of fish should be used to develop SEIR models (i.e., susceptible-exposed-infectious-recovered) specific to fungal pathogens and fish hosts. This would allow a simulation of the true extent of the ecological risk and provide elements for a better environmental monitoring and understanding of these types of pathogens.

### Conflict of interest statement

The authors declare that the research was conducted in the absence of any commercial or financial relationships that could be construed as a potential conflict of interest.
